# Hotspots and determinants of women’s discriminatory attitude towards people living with HIV; evidence from ethiopian demographic and health survey data

**DOI:** 10.1186/s12905-022-01997-3

**Published:** 2022-10-21

**Authors:** Atalay Goshu Muluneh, Mehari Woldemariam Merid, Getahun Molla Kassa, Desalegn Anmut Bitew, Menberesibhat Getie Ferede

**Affiliations:** 1 Department of Epidemiology and Biostatistics, Institute of Public health, college of medicine and Health sciences, University of Gondar, Gondar, Ethiopia; 2 Department of Reproductive health, Institute of Public health, College of medicine and Health sciences, University of Gondar, Gondar, Ethiopia; 3 Departments of Human Anatomy, College of Medicine and Health Sciences, University of Gondar, Gondar, Ethiopia

**Keywords:** Determinants, Discriminatory attitude, Hotspots, PLWH, And Ethiopia

## Abstract

**Background:**

Human Immunodeficiency Virus (HIV) is the major public health concern in Ethiopia with more profound effect on women. Discriminatory attitude towards people living with HIV (PLWH) impose a significant impact on patient outcomes and related issues. Hence, this study aimed to investigate the hotspot areas and determinant factors of women’s discriminatory attitude towards people living with HIV.

**Methods:**

An in-depth secondary data analysis was conducted based on Ethiopian demographic and health survey (EDHS) 2016. A total of weighed 13,822 reproductive-age women were included in the analysis. The non-spatial analysis was conducted using Stata 16. A mixed effect multi-level logistic regression model was fitted to identify determinant factors of discriminatory attitude towards PLWH. A p-value < 0.2 and 0.05 were used as a cut-off point to declare statistical significance for the bi- and multi-variable regression models, respectively. Four separate models i.e. the null, individual, community level model, and a fourth combined model were fitted. Model comparison was done using deviance. Random effect parameters such as correlation coefficient, median odds ratio, and proportional change in variance were used to explain the variation between and within clusters. Global and local level spatial analyses were conducted using Global Moran’s index, GetisOrd Gi* statistics, and Spatial scan statistics were conducted.

**Results:**

The magnitude of women’s discriminatory attitude towards PLWH was 62.66% (95%CI: 60.12, 65.10). The discriminatory attitude of women towards PLWH was spatially clustered (Moran’s index = 0.41, P < 0.01). The hotspots of discriminatory attitude towards PLWH were detected in most parts of the Tigray region; Northern, and southeast borders of the Amhara region; Addis Ababa city; Central, Southern, and western Oromiya region; and East, south, and northeastern parts of South Nations, Nationalities and Peoples Region (SNNPR). Being rural resident, and having no media exposure were positively associated while better educational statuses, better wealth index, unmarried, having comprehensive HIV knowledge, Orthodox religion fellow, and ever being tested for HIV were negatively associated with women’s discriminatory attitude towards people living with HIV.

**Conclusion:**

Discriminatory attitude of women towards PLWH was high in Ethiopia. Hotspots were detected in Amhara, Oromiya, SNNPR, Tigray regions, and Addis Ababa city. Socio-demographic, socio-economic, and HIV knowledge-related factors determine the women’s discriminatory attitude towards PLWH.

## Background

Human immunodeficiency virus (HIV) infection is a medical as well as a social issue[1].Globally, roughly 37.6 million individuals are living with HIV by the year 2020 and around 5000 young women between the ages of 15 and 24 become infected with HIV every week [2].

In Sub-Saharan Africa, new HIV infections account 85%among teenager girls, aged 15 to 19 years [2]. Four thousand five hundred youth population become infected with HIV in Sub-Saharan Africa by the year 2019 accounting for 59% of all new HIV infections in the region [3].

Ethiopia has a concentrated epidemic, with an adult HIV incidence of 0.9% [4]. Among 360,000 reproductive age women (RAW) living with HIV, there were 6,100 newly HIV infected, 6,200 deaths due to AIDS by 2020 in Ethiopia [5].

Although stigma and discrimination associated with HIV/AIDS exist globally, it is a major problem in developing countries where a strong cultural, moral, and religious values are highly practiced across communities [6]. In Ethiopia, there is regional variations in the magnitude of discrimination towards HIV/AIDS reported in Dessie (41.93%) [7], Jimma referral hospital (56%) [8], Oromiya (62%) [9], and Amhara region (34%) [10].

Stigma and discrimination within communities and families has adverse consequences including non-adherence to medications, increased psychological distress; physical and emotional/verbal abuse; low social support, isolation; and risky health behaviors such as medication hiding [11]. Moreover, stigma and discrimination is incriminated as a major barrier for the success of HIV prevention and care programs [6]. As a result, people who are at risk of HIV infection or are unsure if they have it may not be tested for HIV due to fear of being stigmatized and the fear of losing privacy and confidentiality about their HIV status in health-care settings [12, 13]. It also leads to avoidance or postponement of needed care and treatment, as well as poor adherence to antiretroviral therapy (ART) which accelerates disease progression [1, 14]. Indeed, such stigma and discrimination related to HIV significantly contributes to the continuation of the HIV epidemic by reducing HIV testing and preventing undiagnosed HIV-positive patients from receiving essential care [15]. It also has an impact on the quality of life of PLWHA, their families, and the healthcare professionals that work with them face losing their job or income, being isolated from their communities, and being unable to contribute as productive members of society [1].

Important factors influencing discriminatory attitudes towards PLWHA are educational status, economic status, employment status, internet use, residence, media exposure, HIV testing, marital status, region, high-risk taking behavior, individuals associated with stigmatized identities, sources of HIV infection, stage of the disease, and relationship with an infected person [1, 6, 16, 17].

Even though HIV-related programs such as education and stigma reduction, behavior change initiatives, expanded HIV testing, and youth programs [18] have grown significantly, negative attitudes and discriminatory practices towards PLWH remain a major and persistent barriers to compressive HIV care service uptakes [3, 19–21].

Although large numbers of PLWH are found in Ethiopia and discrimination towards PLWH is high, determinants of discriminatory attitudes towards PLWH have not been well addressed. Few available evidences are done locally but none of them are representative at the country level. This study uses a nationally representative data which is possible to generalize to all Ethiopian reproductive age women. One nationally representative study has been done among all people aged 15–49 regardless of sex of respondents. But it didn’t answer what are determinants of the discriminatory attitude of reproductive age women towards PLWH? Therefore, this study aimed to identify the determinants of women’s discriminatory attitudes towards PLWH among reproductive-age women using the 2016 Ethiopian demographic health survey data.

## Methods

### Study area and setting

The study is based on 2016 Ethiopian Demographic and Health Survey (EDHS). Ethiopia is the second most populous country in Africa with more than 110 million populations and where several instabilities existed. Ethiopia is one of the high HIV/AIDS burden countries in Africa.

### Study design and period

We undertake an in-depth secondary data analysis using evidence EDHS 2016 data set. The EDHS 2016 is a nationwide community-based cross-sectional study. The EDHS was based on 645 enumeration areas. Details of the EDHS methodology and measurements were available in the EDHS reports[22].

### Sample size and sampling technique

The national survey used 645 enumeration areas (EAs) as study units. Each EA includes 30 households (HHs)on average. The GPS data was collected as part of the survey and it was collected at cluster (EAs) level [22].From the 645 clusters (EAs), of which 2 EA had missed GPS data, and 22 EAs had zero GPS data which is incorrectly measured and we had excluded them from our analysis. In the meantime 1,314 study participants were excluded because of the missed outcome variable. Additionally, 364 didn’t know whether or not to buy from vendors with HIV, and 263 were not sure whether to allow their children to attend school with HIV-positive children. Moreover, 323 observations were removed because of zero coordinates.

**Fig. 1:**
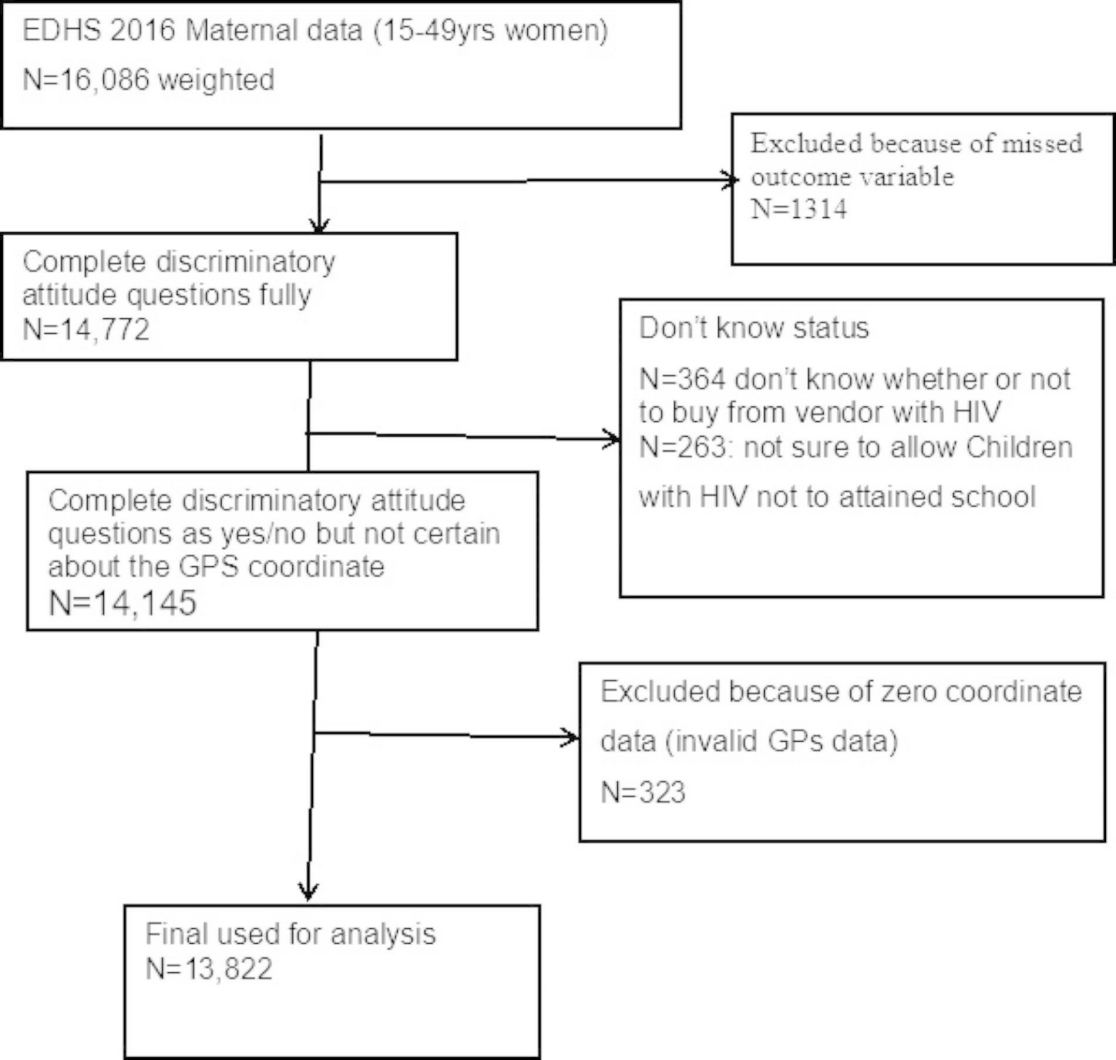
Data extraction process from EDHS 2016 maternal data

Before analysis, we weight the data using the svy Stata command as per the survey guidelines recommendation to balance the differences in response rates. A total of 13,822 weighted sample sizes were used for the final analysis (Fig. 1).

### Variables of the study

The dependent variable was women’s discriminatory attitude towards PLWH reported as yes/no. Independent variables are socio-demographic characteristics like age of the woman, types of residence, educational status of women, media exposure, marital status, religion, and wealth index, and HIV-related factors such as being tested for HIV, and Comprehensive knowledge about HIV[22, 23].

### Operational definitions

#### Women’s discriminatory attitude towards PLWHIV(yes/no)

Reproductive age women with discriminatory attitudes towards people living with HIV are those who say that they would not buy fresh vegetables from a shopkeeper or vendor if they knew that person had HIV, or who say that children living with HIV should not be allowed to attend school with children who do not have HIV[22].

#### Media exposure

Was considered yes, if women read the magazine, use the internet, watch television or listen to the radio at least once a week[22].

#### Comprehensive knowledge about HIV

Was considered as yes, if the women know that consistent use of condoms during sexual intercourse and having just one uninfected faithful partner can reduce the chances of getting HIV, knowing that a healthy-looking person can have HIV, and rejecting the two most common local misconceptions about transmission or prevention of HIV[22].

### Data collection

The survey used pretested and validated standard tools with continuous supervision[22]. For the current analysis, to maintain the data quality, we use the codebooks available from the EDHS 2016 data to extract, clean and recode variables.

### Data analysis

#### Descriptive analysis

we weighed the data before the actual analysis. Proportions and frequencies were used to describe the characteristics of the study participants including figures, tables and narratives.

#### Spatial analysis

The spatial analysis was done using ArcGIS 10.3 and SaTScanTM TM 9.4 software. The presence of spatial autocorrelation at the national level was assessed using the global morals index (Moran’s I statistics). If Moran’s I is greater than zero, we consider spatial clustering while the reverse will be spatial dispersion. After assessing the national level autocorrelation, we used the Getis-Ord Gi* statistics to identify local level spatial autocorrelation/hotspots. The Z-score was used with the null value of -1.96, + 1.96. If the Z-value is greater than 1.96 it refers to a hotspot/high-risk area while the Z-score <-1.96 indicates cold-spot/low-risk areas. Moreover, the spatial scan statistics was used to identify the most likely hotspot clusters. For spatial scan statistics, the default adjustment for the population at risk was 50% but it is also recommended to use less than 50% considering different conditions [24]. We conducted the spatial scan statistics using the Poisson model by considering the scanning window with 25% of the population at risk. Finally, the Log-likelihood ratio (LLR) with a p-value was reported for significant and most likely high-risk clusters of women’s discriminatory attitude toward PLWH in Ethiopia. The hotspot analysis was based on sampled areas only but considering the non-sampled areas too is very important. The spatial interpolation technique using the empirical Bayesian kriging approach was employed to predict high-risk clusters of women’s discriminatory attitudes towards PLWH in Ethiopia by considering the non-sampled areas.

**Multivariable multilevel analysis**: a two-stage mixed effect multilevel logistic regression model was used to determine the individual and community level factors affecting women’s discriminatory attitude towards PLWH and to quantify the cluster variability. We fitted four models. Model I: the null model (empty model) was fitted without any independent variable to estimate the intra cluster correlation (ICC) just to show the extent of intra-cluster variation. Model II (Individual level model): was fitted with only individual-level variables to measure the effects of individual level variables. Model III (community level model): where only community level factors like region, types of place of residence were considered, and the final (Model IV) where individual and community level variables were fitted simultaneously to determine the combined effects of individual and community level variables. The adjusted odds ratio (AOR) with 95%CI was reported for significant variables after adjusting for individual and community level variables. The chi-square and multicollinearity assumptions were tested. A P-value < 0.2, and 0.05 were used to declare the level of significance at the bi-variable and multivariable multilevel logistic regressions respectively.

#### Random effect analysis

The random effects were measured by ICC, median odds ratio (MOR), and proportional change in variance (PCV) [25] The ICC was calculated to evaluate whether the variation in women’s discriminatory attitude towards PLWH is primarily within or between communities. In our article, MOR shows the extent to which the individual probability of women’s discriminatory attitude towards PLWH contributed by the residential area. The PCV was used to quantify the cumulative effect of individual and community level factors on women’s discriminatory attitudes towards PLWH. The deviance (D) was estimated as two times the absolute magnitude of log likelihood to select the better-fitted model. A model with small value of deviance was used as better model to explain the data. The model IV was the better with smallest values of deviance and used for final discussion.

## Results

Nearly two-thirds (62.66%: 95%CI: 60.12, 65.10%) of the women in Ethiopia had discriminatory attitudestowards PLWH.Nearly two-thirds (63.08%)of the women were unmarried. More than three-fourths (77.49%) of women had no comprehensive HIV knowledge. Regarding to the HIV test status, more than half (51.68%) of women were not ever tested (Table 1).

**Table 1:** Individual level characteristics of study participants (weighted N = 13,822)

Variable	Category	Discriminatory attitude		Frequency (%)
		Yes	No	
Age of the respondent	Below 20	1632	1350	2982(21.57)
	20 to 25	1903	1350	3252(23.53)
	26 to 35	2904	1544	4448(32.18)
	Above 35	2223	917	3140(22.71)
Marital status	Married	6158	2561	819 (63.08)
	Notmarried	2503	2600	5103(36.92)
Educational status	No formal education	4943	1191	5103(36.92)
	Primary education	3142	1916	5058(36.59)
	Secondary	471	1292	1763(12.76)
	Above secondary	106	761	867(6.27)
Occupation	Working	4186	2867	7053(51.02)
	Not working	4475	2294	6769(48.98)
Wealth status	Poorest	1668	355	2023(14.63)
	Poorer	1872	481	2353(17.03)
	Middle	1964	650	2614(18.91)
	Richer	1834	978	2812(20.34)
	Richest	1324	2696	4020(29.08)
Ever been tested for HIV	No	5227	1916	7143(51.68)
	Yes	3435	3244	6679(48.32)
Comprehensive HIV knowledge	No	7481	3229	10,470(77.49)
	Yes	1180	1932	3112(22.51)
Media exposure	Yes	1529	2437	3966(28.70)
	No	7132	2723	9855(71.30)

Relating to community-level characteristics, three-fourths (75.65%) of women live in rural Ethiopia. nearly half (45.44%) of the women were orthodox religion followers (Table 2)

**Table 2:** Community level characteristics of study participants (weighted N = 13,822)

Variable	Category	Discriminatory attitude		Frequency (%)
		Yes	No	
Place of residence	Rural	7715	2741	10,456(75.65)
	Urban	947	2420	3366(24.35)
Religion	Orthodox	3274	3007	6281(45.44)
	Muslim	2817	1112	3930(28.43)
	Protestant	2342	1000	3342(24.18)
	Other	228	41	269(1.95)
Region	Tigray	627	456	1083(7.84)
	Afar	62	41	103(0.75)
	Amhara	1954	1454	3428(24.80)
	Oromia	3318	1426	4744(34.32)
	Somali	154	34	188(1.36)
	Benishangul	78	65	143(1.03)
	SNNPR	2248	835	3084(22.31)
	Gambella	15	23	38(0.28)
	Harari	14	21	35(0.25)
	Addis Ababa	163	733	896(6.48)
	Dire Dawa	29	50	79(0.57)

**Fig. 2:**
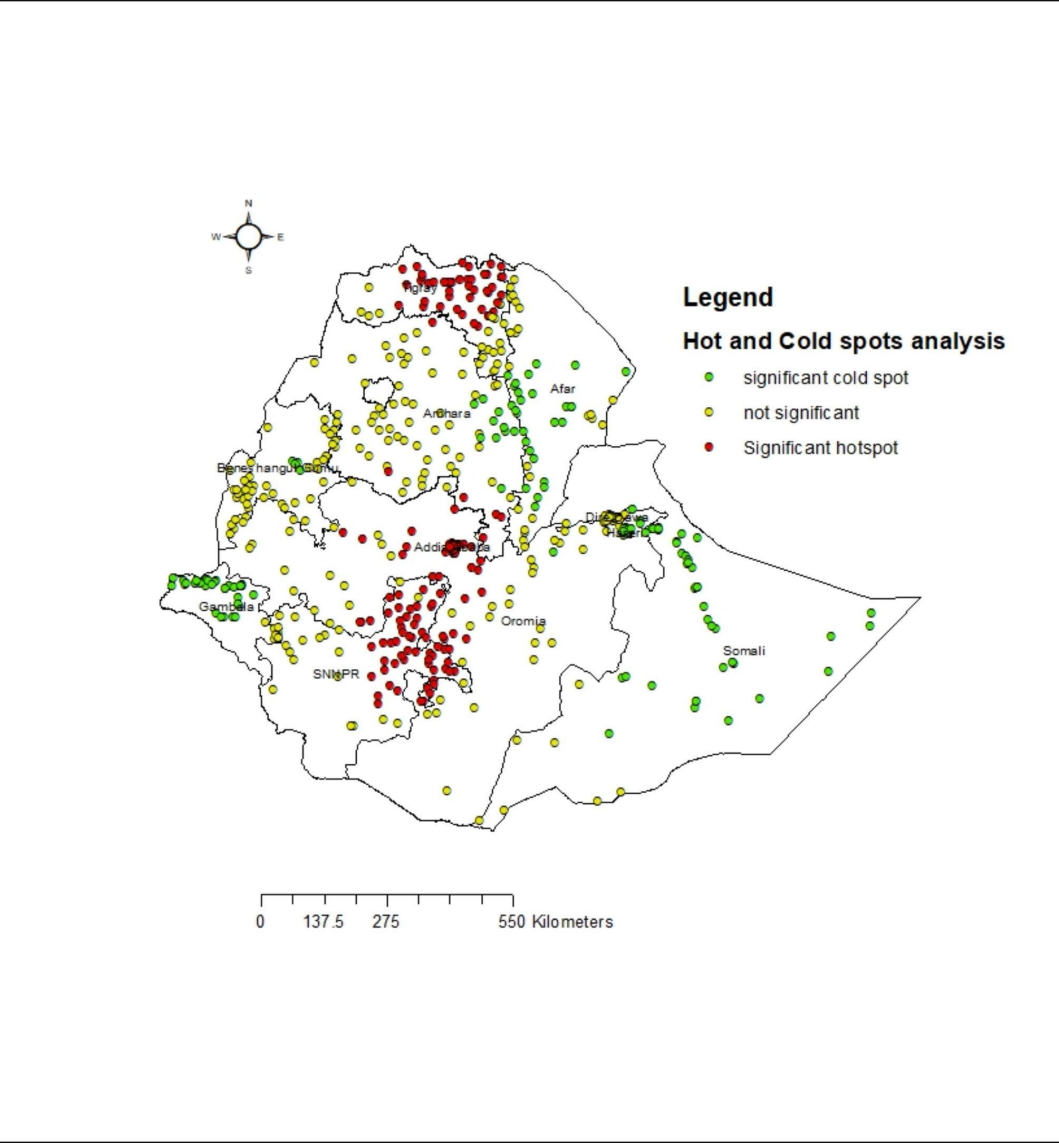
Hotspot analysis of women’s discriminatory attitude towards PLWH, evidence from EDHS 2016. Shapefile from open Africa

**Hotspots of women’s discriminatory attitude**: The distribution of women’s discriminatory attitude was spatially clustered in Ethiopia( Moran’s I = 0.41, P < 0.01). Hence, further spatial analysis techniques were required to detect the local level spatial clusterings (Hotspots) of women’s discriminatory attitudes of women towards PLWH. As shown below by the red dots on the map, hotspots of women’s discriminatory attitudes towards PLWH have been detected in most parts of the Tigray region; Norther, and southeast borders of the Amhara region;Addis Ababa city; Central, Southern, and western Oromia region; and East, south and northeastern parts of SNNPR (Fig. 2)

Most likely clusters were detected indifferent regions.Most likely clusters are primary clusters detected by the spatial scan statistics found within the circle of the primary clusters of analysis. These are clusters with highest RR, LLR and lowest and significant p-value.We aggregate the number of women having discriminatory attitude towards PLWH (a case file), and the number of women who respond no for discriminatory attitude as controls. Then we used the case and control file as impute data sets to conduct the purely spatial scan statistics. The observed number of cases were directly aggregated from the data we impute for analysis in a given primary or secondary clusters while the expected number of cases was generated by the software considering the imputed data through the likelihood estimation approach. Primary and secondary clusters are the most high risk areas that should be considered/need primary attention for intervention. The primary clusters are these areas with the highest risk of discriminatory attitude towards PLWH.

**Fig. 3:**
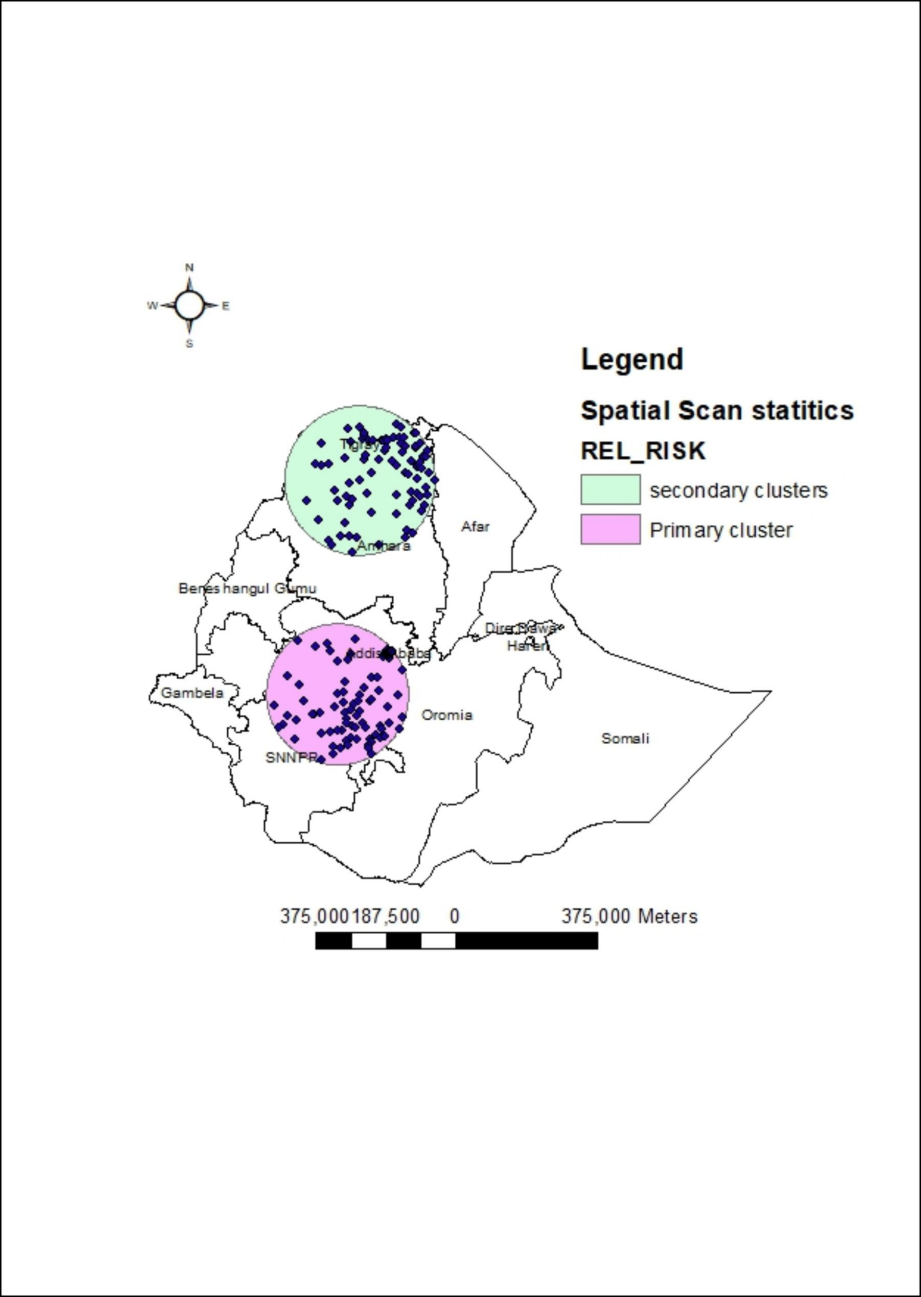
Spatial Scan statisticalanalysis of mostlikely significant clusters of women’s discriminatory attitude towards PLWH, evidence from EDHS 2016. Shapefile from open Africa

The primary high-risk mostlikely clusters were detected in Addis Ababa, Central Oromia, and the Northern part of SNNPR. Secondary clusters were detected inthe Tigray region and the Northern part of the Amhara region. High risk clusters are called hotspot areas. For hotspot analysis using the Get’s Ord Gi* statistics with Z-score > 1.96 is considered as hotspot/high risk areas compared to other clusters (Fig. 3, **&** Table 3). This shows consistent findings with the Geti’s Ord Gi* hotspot analysis.

**Table 3:** summary of mostlikely and high-risk clusters identified using the Spatial Scan statistics

	Enumeration area	Coordinate radius	RR	LLR	population	Observed/expected cases
Primary	142, 174, 577, 502, 262, 223, 331, 272, 359, 271, 227, 41, 204, 297,537, 360, 447, 486, 118, 432, 14, 633, 388, 23, 565, 126, 373, 113, 139, 420, 62, 54, 306, 485, 217, 234, 609, 162, 53, 76, 347, 399, 411, 522, 141, 20, 280, 578, 489, 180, 391, 148, 308, 517, 147, 589,216, 408, 434, 438, 12, 338, 154, 145, 215, 261, 608, 475, 236, 252,586, 83, 353, 645, 487, 314, 110, 365, 225, 59, 195, 539, 451, 61,100, 302, 31, 339, 107, 159, 11, 264, 294, 108, 626, 635, 274, 293,91, 369, 305, 414, 532, 463, 19, 582, 15, 639, 170, 207, 144, 153, 330, 112, 155, 247, 406, 464, 211, 428, 509, 560, 619, 402, 287, 634,90, 290, 26	(8.016479 N, 37.530003 E) / 188.46 km	1.38	129.4	17,347	3553/2775
Secondary	80, 322, 425, 628, 152, 312, 188, 340, 258, 638, 327, 551, 640, 612, 199, 579, 156, 583, 575, 181, 296, 542, 98, 636, 584, 255, 163, 597,538, 400, 504, 528, 590, 81, 424, 66, 355, 268, 78, 253, 392, 84,430, 481, 279, 300, 136, 512, 160, 604, 292, 132, 143, 45, 237, 550, 158, 461, 605, 449, 220, 384, 129, 226, 479, 79, 89, 169, 627,623, 401, 97, 128, 442, 421, 351, 99, 341, 298, 73, 598, 456, 404, 591, 196, 511, 478	(13.159408 N, 38.054771 E) / 199.40 km	1.30	54.97	11,822	2162/1752

**Fig. 4:**
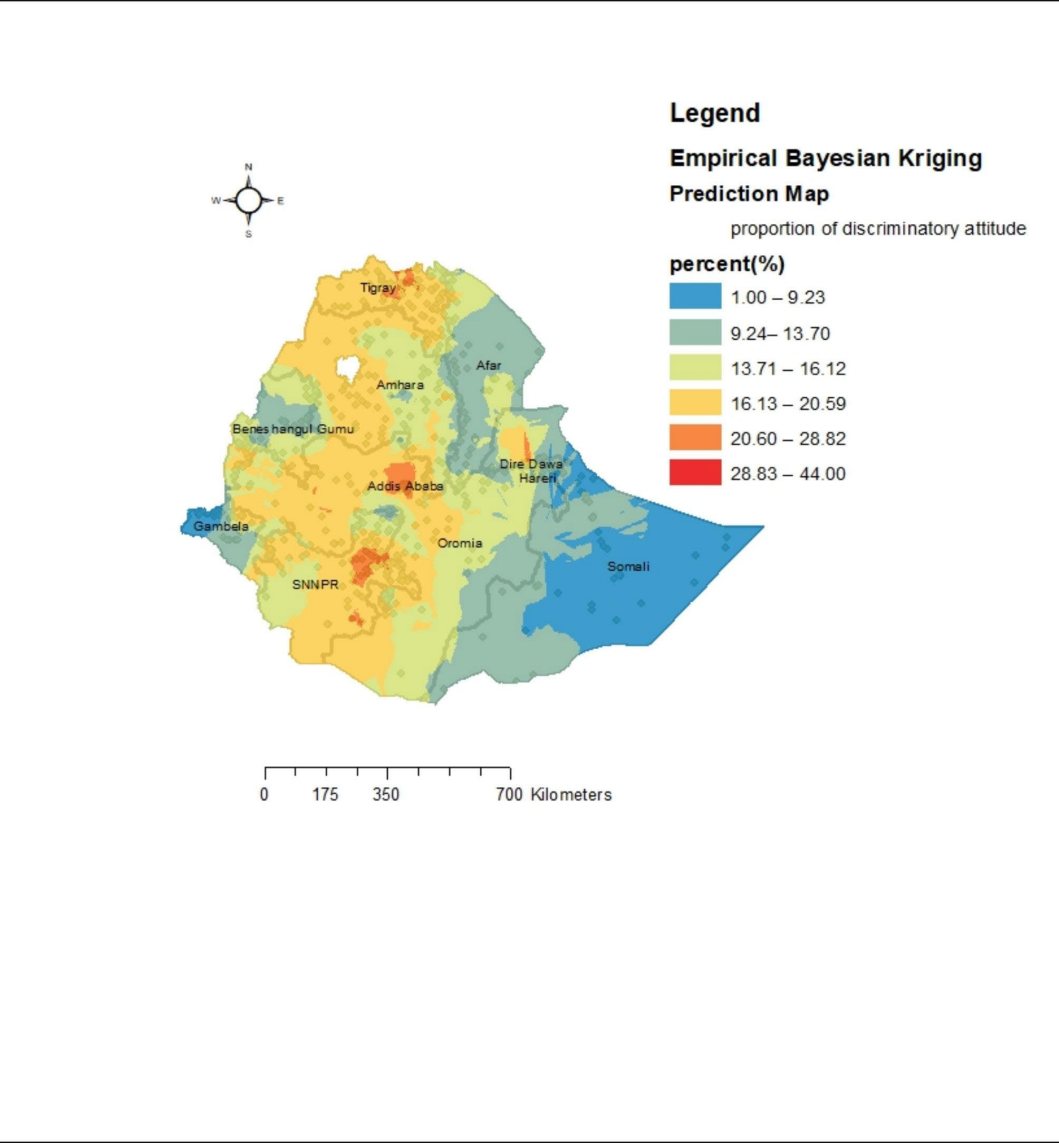
Empirical Bayesian Kriging prediction of Ethiopian women’s discriminatory attitude towards people living with HIV: Evidence from EDHS 2016. Shapefile from open Africa

Empirical Bayesian interpolation was employed to predict the risk of the discriminatory attitude of women towards PLWH. The predicted high-risk clusters were found in the Central, and Northern Tigray region, Addis Ababa city, and South and North Eastern parts of SNNPR with an expected prevalence of 28.83 to 44.00% as stated by the red in the map (Fig. 4) Whereas the cold spots were predicted ineastern Somalia and west Gambela with a predicted prevalence of < 10%.

### The random effect analysis

As evidenced from the empty model, 39.28% (ICC = 0.3928) of variation of the odds of women’s discriminatory attitude towards PLWH was accountedby variations between cluster characteristics. The cluster variability decline successively from 39.28% in the empty model to 14.60, 19.11, and 13.84% in the individual, community level, and final combined models respectively. We found that there was an increased proportion of explained variation in women’s discriminatory attitude towards PLWH as explained by the PCV.i.e 50% increased from the empty model. This implied that 50.00% of the variance in discriminatory attitude towards PLWH was explained by the individual and community level factors together. On top of this, discriminatory attitude towards PLWH was significantly affected by community-level characteristics. Based on the empty model report, the presence of variations between communities was nearly four times (MOR = 4.02) higher than the reference. The unexplained variation in the community decreased to 2.00 in the final model when individual and community level variables were added from the empty model. We compared the model fitness using the deviance, and we found the final model was the best fitted model to explain our data (Table 4**).**

**Table 4:** Random effect models of women descriminatory attitude towardsPLWH, evidence from EDHS 2016

Parameter	Model I	Model II	Model III	Model IV
ICC	0.3928(0.3588, 0.4279)	0.1460(0.1233, 0.1720)	0.1911(0.1659, 0.2192)	0.1384(0.1166, 0.1635)
Variance(sd)	1.46(1.36, 1.57)	0.75(0.68,0.83)	0.88(0.81, 0.96)	0.73(0.66,0.80)
PCV(%)	Reff	48.63	39.73	50.00
MOR	4.02	2.04	2.32	2.00
AIC	15,419	13,877	14,934	13,802
BIC	15,434	13,997	14,979	13,953
LL	-7707	-6922	-7461	-6801
Deviance	15,414	13,844	14,922	13,602

### Fixed effect analysis of determinant factors

Rural resident women had more than two times (AOR = 2.04, 95%CI: 1.64, 2.54) higher odds of discriminatory attitude towardsPLWHcompared to the urban residents.The odds of discriminatory attitude towards PLWH among womenwho had no media exposure was increased by 30% (AOR = 1.30, 95%CI: 1.17, 1.46) as compared to their counterparts.

Compared to protestants, orthodox religion fellow women had more than a third(AOR = 0.67, 95%CI: 0.57, 0.78) fewerodds of discriminatory attitude towards PLWH.Women from the middle, richer and richest households had 18%(AOR = 0.82, 95%CI: 0.69, 0.98), 27%(AOR = 0.73, 95%CI: 0.61, 0.86), and 49% (AOR = 0.51, 95%CI: 0.41, 0.63)lower odds of discriminatory attitude towards PLWHrespectively compared to the poorest households. unmarried women had 25%(AOR = 0.75, 95CI: 0.67, 0.83) lower odds of discriminatory attitude towards PLWH as compared to married women. These women who were ever been tested for HIV had 36%(AOR = 0.64, 95%CI: 0.58, 0.71) lower odds of discriminatory attitude towards PLWH compared to never tested women. Similarly, women who had comprehensive HIV knowledge had 53%(AOR = 0.47, 95%CI: 0.42, 0.52) lowerodds of discriminatory attitude as compared to their counterparts.

Additionally, women who had primary, secondary, and above secondary education had 42%(AOR = 0.58, 95%CI: 0.51, 0.65), 72%(AOR = 0.28, 95%CI: 0.24, 0.33) and 82%(AOR = 0.18, 95%CI:0.14, 0.23) lower odds of discriminatory attituderespectively compared to women with no formal education (Table 5).

**Table 5:** Multilevel mixed effect logistic regression analysis of women’s descriminatory attitude towardsPLWH, evidence from EDHS 2016 data

Variable	Category	Model 1 (AOR, 95%CI)	Model 2(AOR, 95%CI)	Model 3(AOR, 95%CI)	Model 4(AOR, 95%CI)
Empty	Null	1.48(1.30, 1.67)			
Age of the respondent	Below 20	N/A	1.10(0.94, 1.28)	N/A	1.06(0.90, 1.23)
	20 to 25	N/A	1.15(1.01, 1.32)	N/A	1.12(0.98, 1.29)
	26 to 35	N/A	0.98(0.87, 1.11)	N/A	0.98(0.86, 1.10)
	Above 35	N/A	Reff	N/A	Reff
Marital status	Married	N/A	Reff	N/A	Reff
	Notmarried	N/A	0.71(0.64, 0.79)	N/A	0.75(0.67, 0.83)**
Educational status	No formal education	N/A	Reff	N/A	Reff
	Primary education	N/A	0.57(0.51, 0.64)	N/A	0.58(0.51,0.65)**
	Secondary	N/A	0.27(0.23, 0.32)	N/A	0.28(0.24, 0.33)**
	Above Secondary	N/A	0.17(0.14, 0.21)	N/A	0.18(0.14, 0.23)**
Wealth status	Poorest	N/A	Reff	N/A	Reff
	Poorer	N/A	0.90(0.76, 1.06)	N/A	0.93(0.78, 1.10)
	Middle	N/A	0.79(0.66, 0.94)	N/A	0.82(0.69, 0.98)**
	Richer	N/A	0.68(0.57, 0.81)	N/A	0.73(0.61, 0.86)**
	Richest	N/A	0.33(0.27, 0.39)	N/A	0.51(0.41, 0.63)**
Ever been tested for HIV	No	N/A	Reff	N/A	Reff
	Yes	N/A	0.62(0.56, 0.69)	N/A	0.64(0.58, 0.71)**
Comprehensive HIV knowledge	No	N/A	Reff	N/A	Reff
	Yes	N/A	0.46(0.41, 0.52)	N/A	0.47(0.42, 0.52)**
Media exposure	Yes	N/A	Reff	N/A	Reff
	No	N/A	1.35(1.21, 1.52)	N/A	1.30(1.17, 1.46)*
Place of residence	Urban	N/A	N/A	Reff	Reff
	Rural	N/A	N/A	8.00(6.70, 9.35)	2.04(1.64, 2.54)*
Religion	Orthodox	N/A	N/A	0.68(0.58, 0.80)	0.67(0.57, 0.78)**
	Muslim	N/A	N/A	1.25(1.06, 1.48)	0.87(0.73, 1.03)
	Protestant	N/A	N/A	Reff	Reff
	Other	N/A	N/A	1.67(1.10, 2.53)	1.43(0.92, 2.21)

** statistically significant protective factors, * statistically significant risk factors, N/A: not applicable at that level, Reff: reference category

## Discussion

This study aimed to assess the hotspots and determinants of women’s discriminatory attitudes towards people living with HIV. Nearly two-thirds (62.66%: 95%CI: 60.12, 65.10%) of reproductive-age women in Ethiopia had a discriminatory attitude towards PLWH. This finding was lower than the study conducted among the general population of Ethiopia (62.66 vs. 74.7%) [26] which might be explained by variation in a population where the previous study was among the general public including males and discriminatory attitude was more common in males where discriminatory attitude was 93.8% and 64.5% among males and females, respectively which might have inflated the result in the previous study [27].On the other hand, our finding was higher than a study conducted in Iran [28]. This variation might be due to the difference in the population where the Iran study was on the general public in train stations with better education and socio-economical statuses.

The significant hotspots of discrimination were found in most parts of the Tigray region; Northern, and southeast borders of the Amhara region; Addis Ababa city; Central, Southern, and western Oromia region; and East, south, and northeastern parts of SNNPR. We could not find similar reports to compare.

In our study, rural residents had more than two times higher odds of discriminatory attitudes as compared to urban residents. This finding is supported by other studies conducted in Ethiopia [26, 29], Tajikistan [30], and china [31] which shows individuals who lived in rural residences were more likely to show stigma and discriminatory attitudes compared with their counter parts. Women who are living in urban have access of mass media like television, radio, newspaper, which favor their access for health promotion messages and particularly for stigma- and discrimination-related messages. In addition to this, urban women are more likely to be educated and economically empowered. As result they can have high comprehensive knowledge about HIV/AIDS. These collectively will reduce negative attitude towards PLWH [32].

Women who were not exposed to media had 1.3 times more odds of discriminatory attitudes as compared to those exposed. This is supported by other findings from Ethiopia [1, 26] which might be explained by the fact that women with better media access may have better comprehensive HIV knowledge which can prevent them to avoid unnecessary behaviors like discrimination and stigma and dilutes pre-existing misconceptions regarding HIV/AIDS [1, 33].

Regarding the educational status, women with formal education had fewer odds of discriminatory attitudes towards PLWH. This is supported by other studies findings from Ethiopia [1, 26, 34], Kenya [35],and Nigeria [36] where better educational status was negatively associated with discriminatory attitudes towards PLWH. This might be justified by those individuals with better education who may have access to better information through mass media, the internet, and access to health services related to HIV/AIDS and that will help them to care for and be compassionate for PLWH [1].

On the other hand, married individuals have a more discriminatory attitude toward HIV/AIDS than unmarried women. This is comparable with studies done in Ethiopia [1],

China [37] and Nigeria [36]. It might be due to most married people in Ethiopia living in rural settings and have not attended school to a high level and they might not access the internet, television, and radio due to workload and/or living arrangements [1].

Orthodox religion fellow had less odds of discriminatory attitude compared to Protestants. This is supported by other studies finding from Thai where the Muslim religion fellow had more odds of discriminatory attitude to PLWH [15]. It is evidenced by one study finding on several religious scales and measures of discriminatory attitudes toward blacks, women, homosexuals, and communists, Christian orthodoxy and intrinsic religious orientation were negatively related to these variables [38].

Women with middle, rich, and richest household wealth index had lower odds of discriminatory attitude as compared with the poorest. This is comparable with findings from Thai, Indonesia [39], and Malaysia [17].This might be due to rich people may have better knowledge of HIV, and accessing different behavioral change communications through mass media or social media [40].

Related to HIV-related conditions, women who were ever tested for HIV had lower odds of discriminatory attitudes about PLWH. This is supported by study findings from Ethiopia [26]. Individuals might gain HIV-related information during counseling at the time of HIV testing which may reduce negative attitudes towards HIV-infected people [41].

Women with comprehensive HIV knowledge had lower odds of discriminatory attitudes against PLWH. This was supported by findings from Ethiopia [26], this may be explained by individuals who have no comprehensive HIV knowledge may have misconceptions about modes of transmission and may have a poor understanding of the consequence of discriminatory attitudes [42].

## Strengths and limitations

We believe our study had several strengths such as we used nationwide data with better statistical power, and using spatial and multilevel approaches. We don’t hide that the geographic distortion of coordinate data for security purposes may affect the spatial analysis. Additionally, we rely on the available secondary data and important cultural practices, and their HIV statuses were not included. But accuracy of the data could be affected by recall bias since the source of the data was self-report. In addition to this using secondary data limit the researcher to measure all possible determinants like culture and tradition related factors.

## Conclusion

Discriminatory attitude of women toward PLWH was high. Hotspots of discriminatory attitudes were identified in Addis Ababa, Oromiya, SNNPR, and Tigray regions of Ethiopia. Different socio-demographic and economic characteristics significantly affect the women’s discriminatory attitude towards PLWH in Ethiopia. Our results suggest that strategies to reduce discriminatory attitudes towards PLWH are needed and very important information, education and communication programmes focusing on stigma and discrimination are needed to reduce misconceptions. Economic empowerment of women should also be considered. In addition, expanding access to HIV counseling and testing services should be done.

## Data Availability

The data used for the preparation of this manuscript are available from http://www.dhsprogram.comand anyone can access it through an online request as an authorized user. The authors prepared the data that was used for the preparation of this manuscript can be shared if required.

## Abbreviations

AOR: adjusted odds ratio.
EDHS: Ethiopian Demographic and Health Survey.
AIC: Akakie information criterion.
ICC: Intra class correlation.
DIC: deviance information criteria.
MOR: Median Odds Ratio.
PCV: percentage change in variance.
PLWH: people living with HIV
EAs: Enumeration Areas
RR: Relative Risk
LLR: log likelihood ratio.
